# Medical staff contributions to thirdhand smoke contamination in a neonatal intensive care unit

**DOI:** 10.18332/tid/106116

**Published:** 2019-04-24

**Authors:** Thomas F. Northrup, Angela L. Stotts, Robert Suchting, Amir M. Khan, Charles Green, Penelope J. E. Quintana, Eunha Hoh, Melbourne F. Hovell, Georg E. Matt

**Affiliations:** 1 Department of Family and Community Medicine, McGovern Medical School, The University of Texas Health Science Center at Houston (UTHealth), Houston, United States; 2 Department of Psychiatry and Behavioral Sciences, McGovern Medical School, The University of Texas Health Science Center at Houston (UTHealth), Houston, United States; 3 Department of Pediatrics, McGovern Medical School, The University of Texas Health Science Center at Houston (UTHealth), Houston, United States; 4 Center for Clinical Research and Evidence-Based Medicine, McGovern Medical School, The University of Texas Health Science Center at Houston (UTHealth), Houston, United States; 5 Division of Environmental Health, School of Public Health, San Diego State University, San Diego, United States; 6 Center for Behavioral Epidemiology and Community Health, School of Public Health, San Diego State University, San Diego, United States; 7 Department of Psychology, San Diego State University, San Diego, United States

**Keywords:** thirdhand smoke, THS, environmental tobacco smoke, NICU, medical staff

## Abstract

**INTRODUCTION:**

Non-smoking policies are strictly enforced in neonatal intensive care units (NICUs), which may still become contaminated by thirdhand smoke (THS), posing potential health risks to medically fragile infants. Study aims were to explore contamination routes by characterizing nicotine levels (THS proxy) found on the fingers of NICU medical staff and to assess finger-nicotine correlates.

**METHODS:**

NICU medical staff were surveyed regarding smoking and electronic nicotine devices (ENDS) use/exposure, and household characteristics. Approximately 35% of staff were randomly selected for a finger-nicotine wipe. Three separate quantile regressions modeled percentiles associated with: presence of any finger nicotine, finger-nicotine levels above the median field blank level (i.e. 0.377 ng/wipe), and finger-nicotine levels two times the median blank.

**RESULTS:**

The final sample size was 246 (n=260 approached; n=14 refusals). Over three-quarters (78.5%) reported some exposure to tobacco smoke or ENDS vapor/aerosols. After field-blank adjustments, the median nicotine level (ng/finger wipe) was 0.232 (IQR: 0.021–0.681) and 78.3% of medical staff had measurable finger-nicotine levels. Both being near smoking in friends’/family members’ homes and finger-surface area were related to elevated finger-nicotine levels (p<0.05) in the median blank model.

**CONCLUSIONS:**

Almost four in five NICU staff had measurable finger nicotine, with finger surface area and frequency of reported exposure to tobacco smoke in friends’/family members’ homes emerging as important correlates. Future research will determine the impact of THS on NICU infants. Medical personnel working in a NICU should be cognizant of secondhand smoke and THS, particularly inside friends’/family members’ homes, to reduce potential NICU contamination and infant exposures.

## INTRODUCTION

Aged secondhand smoke (SHS) lingers indoors and also forms a persistent residue, known as thirdhand smoke (THS), which has emerged as a distinct public health hazard^1-5^. Non-smoking policies are strictly enforced in hospital settings, like the neonatal ICU (NICU), but these environments may still become contaminated by nicotine and THS, as shown in our prior work^6^. Exploring routes of contamination and exposure is critical for understanding the potential role of THS exposure in health outcomes for medically fragile pediatric patients exposed during extended hospitalizations.

THS residue and particles transported on hands/skin, hair, clothes, and other objects can be transferred to new surfaces^7-9^, such as NICU incubators and furniture^6^. THS can remain on surfaces and dust and be slowly re-emitted into the gas phase^3,4,10,11^. Infant exposure may take place^6^ via dermal exposure, ingestion, and inhalation of airborne constituents^12,13^. Visitors and employees who smoke, extinguish cigarettes outside and then immediately enter the hospital, will continue to expel particulate matter for up to 90 seconds^14^. Further, their breath and clothing have higher concentrations of neurotoxic/cilatoxic/other toxic substances (e.g. adversely affecting lung cilia) for up to 10 minutes^15^.

Animal and *in vitro* assay studies have demonstrated THS causes damage to DNA^16^, impairs wound healing^17^, and hinders respiratory development in unborn, premature rat fetuses^18^. Data suggest that children only exposed to THS (i.e. no SHS exposure) have more respiratory symptoms than non-exposed children^1^. Increased research has been recommended to determine the degree to which THS harms human health^7,19-21^, especially in children who are more susceptible to THS and its consequences^13^. Health-related findings are particularly concerning for premature, low-birth-weight infants, already at elevated risk for respiratory harm due to higher respiration rates, immaturity of respiratory functioning, low metabolic capacity, and immature or compromised immune systems. Moreover, we do not yet know the extent of health effects currently attributed to SHS, which may be the result of the cumulative effects of chronic THS exposure^21^.

While parents and other visitors are most likely to transport THS to the NICU^22^, medical providers who smoke or who are in smoking environments may also contribute to THS contamination. From 2003 to 2011, data support that physicians (≤2.3%) and registered nurses (≤11.1%) engage in lower smoking rates, compared to the US overall population, whereas respiratory therapists engage in smoking levels closer to the US population (<19.3%)^23-25^. Data are limited regarding the proportion of medical staff who use electronic nicotine devices (ENDS), live with a smoker, or spend time in environments where smoking occurs. The primary aim of this study was to characterize the level of nicotine (a proxy of THS) found on the fingers of NICU medical staff and to assess self-reported smoking and exposure to SHS/THS outside the hospital. Our *a priori* hypotheses were that >10% of medical staff would have measurable levels of nicotine on their fingers and that staff in greater contact with SHS/THS would have higher finger-nicotine levels. Variables related to cigarettes/ENDS use and exposure to cigarettes/ENDS, with a potential for direct influence on finger-nicotine levels, were also investigated. This is the first study to explore finger-nicotine levels with medical staff and adds unique information about NICU staff exposure to SHS/THS.

## METHODS

### Participants and procedures

Participants were medical staff recruited from a large, urban children’s hospital in Houston, Texas with a 144-bed NICU and over 1000 admissions per year. Recruitment to complete a 5-minute survey took place over a 30-day period (beginning in January 2017), in employee lounges and conference rooms. Research staff attended regularly scheduled NICU-staff meetings (e.g. morning/evening rounds) and verbally consented medical staff, before completing any research procedures, in compliance with our institutional and hospital IRBs (HSC-MS-15-0614).

Convenience sampling at staff meetings continued within each medical specialty until ≥60% of staff within each specialty were approached (N=260 approached; 246 consented). Approximately 35% of surveys were randomly marked with a red ‘X’ in the upper-right corner (n=92), indicating selection to complete a nicotine wipe on their thumb, index, or middle finger (dominant hand; n=84 participants wiped). One finger per participant was wiped in sequential fashion across participants: thumb, index, middle and then the order was repeated. These fingers were selected for wiping as a majority of smokers use these fingers when smoking, these fingers are also heavily involved in fine motor activities related to work in the NICU, and methodologically little is known about the distribution of nicotine across the hand. Research and medical staff were blinded to random selection for wiping procedures, as an opaque study information sheet (for medical staff to keep) was clipped to the top of each survey. Research staff handed the paper-clipped pages to medical staff, peeled back the information sheet, and informed the medical staff if they had been randomly selected for a finger wipe.

### Measures

THS surface-nicotine (finger) wipe procedures have been established^8,26-28^. Taking a sample of THS surface nicotine involves: 1) preparing a solution of distilled water and 1% ascorbic acid (i.e. vitamin C); 2) wetting a screened cotton wipe with the solution; 3) wiping the entire surface of the assigned finger (thumb, index, middle); 4) measuring the length and circumference of the finger (for surface area standardization, i.e. treating a finger as a cylinder without a bottom); and 5) storing the wipe in a vial for further analysis. Surface nicotine was quantified as previously published^29^. Surface nicotine levels are reported as the total amount (ng) per finger (consistent with previous studies^26^). Following the recommendation of Quintana et al.^28^, field blanks were collected during every sampling occasion (at the start and end of sampling by each staff member designated to perform finger wipes), which involved following surface wipe procedures with the exception of step 3 (the cotton was exposed to the air but not used to wipe a finger). Consistent with prior work, we analyzed over a quarter of blanks (28.6%). The median blank value was 0.377 ng/wipe (IQR=0.263–0.453). Over half of participants’ finger wipes (57.1%) were able to be associated with a blank collected on the same sampling occasion, which was used to correct (subtract out) nicotine present in the sampling materials and environment from the finger levels. Participant finger wipes not associated with a specific blank (42.9%) were adjusted by subtracting the median blank wipe (0.377 ng) from participant finger wipes.

The survey included questions related to participant characteristics (i.e. sex, age, race/ethnicity, relationship status), household characteristics (i.e. number of adults ≥18 years old), and smoking-related and ENDS-related behaviors. We assessed participant smoking and ENDS use with two separate multiple-choice questions^6,22^. Responses were collapsed to current, former, and never smoked due to very few current smokers. Very few participants reported any household smoking (or ENDS use). Therefore, the total number of smokers (or ENDS users) living in the home was collapsed to 0 and ≥1, and the total cigarettes used per day (regardless of location) by all household members was collapsed to <10 cigarettes/day and ≥10 cigarettes/day, representing light and heavy smoking households^30,31^.

A multiple-choice question was used to assess cigarette smoking and (separately for ENDS) bans in participant: a) homes or b) cars. Similar to our other work, we dichotomized bans so that only a report of a total ban (i.e. ‘no one is allowed to [smoke/use ENDS] in your [home/car] with NO exceptions’ was treated as a ban)^22,32,33^.

Participants were asked an author-constructed question, ‘How often are you near someone smoking?’ in: a) ‘friends/family home(s)’ or b) ‘in other locations’. Response options were: ‘daily or nearly every day’, ‘weekly’, ‘monthly’, ‘less than monthly’, and ‘never.’ The same question and response format was used for assessing ENDS exposure. The responses were recoded to any exposure (i.e. ≥ less than monthly) versus never for the statistical analyses.

### Statistical analyses

Due to positively skewed distributions of finger-nicotine-wipe values, we reported medians and interquartile ranges (IQR). All analyses for the 84 participants with finger-wipe data were conducted in R, version 3.5.1^34^, and all statistical tests were evaluated at the alpha (2-tailed) 0.05 level. Given that current nicotine-contamination thresholds for THS-related harm are not established, quantile regression allowed empirical modeling of three increasingly conservative thresholds in this sample. This methodology provides a starting point for developing consensus thresholds of potential harm in this field. For the highly skewed finger-wipe data, three separate quantile regressions modeled the percentile (tau) associated with: a) presence of any finger nicotine (>0.00 ng/wipe [limit-of-quantification (LOQ)]; tau=0.217), b) finger-nicotine levels above the median blank (i.e. 0.377 ng/wipe; tau=0.587); and c) finger-nicotine levels two times (2×) the median blank (i.e. 0.754 ng/wipe; tau=0.774). In the absence of clinical guidance on safe THS exposure levels, these three thresholds were chosen to correspond to the lowest LOQ, along with two relatively more conservative levels, respectively. The quantile regressions (quantreg package, version 5.36^35^) utilized a two-step exploratory model-building approach whereby eleven theory-chosen predictors were: 1) analyzed in univariate models, and then 2) included in a multiple predictor model if meeting a relaxed threshold of significance (p<0.50) in step one.

## RESULTS

Medical staff participated at high rates (N=260 approached; N=246 participated; 5.4% refusal rate [n=14]). A majority of participants were nursing staff (n=170; 65.6%), the most numerous NICU specialty, followed by respiratory therapists (n=51; 19.7%), fellows/residents (n=14; 5.4%), nurse practitioners (n=12; 4.6%), and physicians (n=12; 4.6%). The mean age of the participants was 36.0 years (SD=10.4), and the sample was predominantly White, non-Hispanic (n=151; 62.4%), followed by Hispanic/Latino (n=35; 14.5%), Black/African-American (n=34; 14.1%), and 7.9% (n=19) were Asian or other races/ethnicities (n=3; 1.2%). Full sample characteristics are shown in Table 1.

**Table 1:** NICU-based medical staff characteristics and self-reported tobacco/ENDS use from 2017

*Characteristics*	*n (%)*
**Total approached^a^**	260 (100.0)
**Refusals^b^**	14 (5.4)
**Female**	215 (87.4)
**Race/ethnicity**	
White, non-Hispanic	151 (62.4)
Hispanic	35 (14.5)
Black/African-American	34 (14.1)
Asian	19 (7.9)
Other	3 (1.2)
**Relationship status**	
Married	139 (56.5)
Single	64 (26.0)
Living together but not married	27 (11.0)
Divorced/separated/widowed	16 (6.5)
**Specialty^c^**	
Nursing	170 (65.6)
Respiratory therapist	51 (19.7)
Fellow/resident	14 (5.4)
Neonatal nurse practitioner	12 (4.6)
Physician	12 (4.6)
**Participants selected for finger wipe^b^**	92 (35.4)
Selected for and consented to wipe	84 (32.3)
**Shift assessment time**	
Beginning of shift (<1 hour after start)	203 (78.7)
Middle of shift (hours 1–11)	31 (12.0)
End of shift (>11 hours after start or shift ended)	24 (9.3)
**One or more smokers reported in home**	15 (6.4)
<10 cigarettes/day by any household member	6 (2.6)
≥10 cigarettes/day by any household member	5 (2.2)
**One or more ENDS users reported in home**	3 (1.4)
**Smoking status**	
Current smoker	3 (1.2)
Former smoker	22 (9.1)
Never smoker/fewer than 100 cigs/lifetime	216 (89.6)
**ENDS status**	
Current ENDS use	0 (0.0)
Former ENDS use	15 (6.2)
Never used ENDS	226 (93.8)
**Any cigarette users reported in the home**	17 (6.9)
**Any cigarette or ENDS users reported in the home**	19 (8.1)
	***M (SD)***
**Participant age** (years)	36.0 (10.4)
**Number of adults** ≥**18 years old in home**	1.9 (1.1)
**Finger surface area (cm^2^)^d^**	
Thumb (n=28)	43.2 (6.9)
Index (n=28)	49.8 (6.2)
Middle (n=28)	53.5 (7.9)
	***Median (IQR)***
**Finger-nicotine wipes (ng/finger)^e^**	0.232 (0.021–0.681)
Thumb (n=28)	0.169 (0.000–0.431)
Index (n=28)	0.232 (0.000–0.769)
Middle (n=28)	0.470 (0.087–0.807)

Data were collected over a 30-day period (beginning in January 2017). Where categories do not add up to 246, the remainder represent missing data.

a One participant did not return their survey and their specialty was not recorded.

b Of the 14 refusals, eight of them had been randomly selected for finger wipes and may have refused for that reason.

c Based on hospital administration data, 61.8% of nurses, 60.0% of respiratory therapists, 70.6% of nurse practitioners, 60% of physicians, 90.9% of fellows, and 100.0% of residents/medical students were approached to participate.

d Finger-surface area varied significantly across finger types (p<0.0001).

e Finger-nicotine levels did not vary significantly across finger types (p=0.22).

Based on self-report, the overwhelming majority of participants did not live with smokers (93.6%) or ENDS users (98.6%), did not smoke cigarettes currently (98.8%), did not use ENDS currently (100%), and banned smoking in their homes and cars, with slightly fewer participants reporting ENDS bans in their homes and cars (Table 2). Participants reported greater exposure to smoking than ENDS, particularly in locations other than friends’/family members’ homes (Table 2). Overall, over three-quarters of the sample (78.5%), reported at least some exposure to tobacco smoke or ENDS vapor/aerosols.

**Table 2:** NICU-based medical staff self-reported secondhand smoke and ENDS vapor exposure, and home or car smoke/vapor ban policies from 2017

*Characteristics*	*n (%)*			

	*Near Smoking*		*Near ENDS*	

*How often near (smoking/ENDS)*	*Friends’/Family members’ homes*	*Other locations*	*Friends’/Family members’ homes*	*Other locations*
Daily or nearly every day	9 (3.8)	5 (2.2)	2 (0.8)	0 (0.0)
Weekly	9 (3.8)	34 (14.7)	4 (1.6)	15 (6.4)
Monthly	11 (4.6)	41 (17.7)	10 (4.1)	32 (13.6)
Less than monthly	44 (18.4)	91 (39.2)	33 (13.5)	69 (29.2)
Never	166 (69.5)	61 (26.3)	195 (79.9)	120 (50.9)
***Ban (smoking/ENDS) in home or car***	***Ban Smoking***	***Ban ENDS***		
Home ban on (smoking/ENDS) indoors	234 (97.5)	224 (93.3)		
Car ban on (smoking/ENDS) indoors	232 (97.5)	228 (95.0)		

Data were collected over a 30-day period (beginning in January 2017). Where categories do not add up to 246, the remainder represent missing data.

The median nicotine was 0.232 ng/finger wipe (IQR: 0.021–0.681) (Table 1). A majority of medical staff (78.3%) had measurable levels of nicotine on their fingers. As a reminder, all finger-nicotine wipes were adjusted by subtracting the level of nicotine found in field blanks, to account for nicotine levels present in the sampling materials and the work environment. Further, 41.3% and 22.6% of staff had finger-nicotine levels above the level of the median blank and two times the median blank level, respectively.

No variables were associated with whether participants’ fingers had any measurable nicotine (>LOQ ng/finger); perhaps not surprising given that almost 80% of staff had measurable levels (Table 3). Being near smoking in friends’/family members’ homes and finger surface area were both statistically significant (p<0.05) in the multiple-predictor model exploring median blank (0.377 ng/wipe; percentile [tau=0.587]). We examined the magnitude of finger-nicotine levels across those reporting exposure and those not reporting exposure in friends’/family members’ homes and found that the median nicotine/finger was greater for participants who reported being near smoking in friends’/family members’ homes (median=0.600 [tau=0.691]; IQR: 0.224–1.088 ng/finger wipe) compared to those who did not (median=0.155 [tau=0.405]; IQR: 0.000–0.500 ng/finger wipe). Further, as finger-surface area increased, finger-nicotine levels increased as well. For the third quantile regression, when twice (2×) the median blank (0.754 ng/wipe) percentile (tau=0.774) was modeled, finger surface area was the strongest predictor but did not reach statistical significance (p=0.11).

**Table 3:** Results of three quantile regressions of finger-nicotine-level percentile (tau) for NICU-based medical staff from 2017

	*Univariate Modeling Results*					
	*Any measurable amount (>0.00 ng/wipe)*		*>Median blank (>0.377 ng/wipe)*		*>2 × Median blank (>0.754 ng/wipe)*	
	*Percentile (tau=0.217)*		*Percentile (tau=0.587)*		*Percentile (tau=0.774)*	
*Characteristics*	*Coefficient (95% CI)*	*p*	*Coefficient (95% CI)*	*p*	*Coefficient (95% CI)*	*p*
Finger surface area^b,c^	0.001 (-0.003–0.006)	0.54	**0.015 (-0.001–0.03)**	**0.07**	**0.045 (-0.009–0.099)**	**0.11**
Number of adults in home	0.007 (-0.036–0.05)	0.76	0.098 (-0.082–0.279)	0.29	0.07 (-0.362–0.503)	0.75
Participant age^b^	-0.001 (-0.003–0.002)^d^	0.76	**-0.007 (-0.019–0.005)**	**0.26**	-0.013 (-0.073–0.047)	0.68
Any cigarette users in home	0.58 (-19.563–20.723)	0.96	0.542 (-57.862–58.946)	0.99	28.578 (-80.702–137.859)	0.61
Any nicotine users in home^a^	**0.178 (-0.323–0.679)**	**0.49**	0.38 (-45.078–45.837)	0.99	28.514 (-69.122–126.15)	0.57
Near smoke in friends’/family members’ homes^a,b^	**0.171 (-0.03–0.372)**	**0.10**	**0.437 (0.017–0.857)**	**0.04**	0.746 (-5.994–7.486)	0.83
Near smoke in any other locations	0.001 (-0.089–0.09)^d^	0.99	0.238 (-0.232–0.709)	0.32	0.264 (-2.681–3.21)	0.86
Participant=lifetime smoker	0.103 (-0.825–1.031)	0.83	1.164 (-56.611–58.94)	0.97	28.578 (-68.655–125.812)	0.57
Participant=female	0.001 (-0.556–0.556)^d^	1.00	-0.404 (-2.181–1.373)	0.66	-1.695 (-66.104–62.713)	0.96
Total smokers in home	0.58 (-37.891–39.051)	0.98	0.319 (-88.802–89.441)	0.99	0.076 (-132.323–132.476)	1.00
Sample time	-0.024 (-0.108–0.061)	0.59	-0.119 (-0.566–0.327)	0.60	0.151 (-1.009–1.311)	0.80

Data were collected over a 30-day period (beginning in January 2017). Variables retained in a final multivariable model appear in boldface (above). Sample time values were: beginning (<1 hour after start of shift), middle (1–11 hours after start of shift), and end (shift ended or >11 hours since start).

a Variables/characteristics retained in the final multivariate model for ‘any measurable amount’. Neither ‘any nicotine users in home’ (p=0.38) or being near smoking (p=0.11) reached statistical significance.

b Variables/characteristics retained in the final multivariate model for ‘>median blank’. Finger area and being near smoking in friends’/family members’ homes reached statistical significance (p<0.05); participant age did not reach statistical significance (p=0.16)

c Finger area was the only variable/characteristic retained in the final multivariate model for ‘>2 × median blank’ but did not reach statistical significance (p=0.11).

d Actual value was less than -0.001 (i.e. participant age) or 0.001 (i.e. near smoke in any other locations and Participant=female).

## DISCUSSION

Similar to our previous work^6^, these data underscore the difficulty of completely removing nicotine contamination, even from the fingers of non-smoking medical staff working in a smoke-free hospital setting. Indeed, almost four in five medical staff members providing care to vulnerable infants had measurable nicotine found on one of their fingers, well above our *a priori* hypothesis of 10% of NICU medical staff having measurable finger-nicotine contamination. We also found two correlates of finger-nicotine levels: finger surface area and frequency of reported exposure to tobacco smoke in friends’/family members’ homes.

Previous research has demonstrated that nicotine found on fingers of non-smokers is associated with staying in hotels that allow smoking^26^, living in homes where a smoker quit months earlier^36^ or was previously occupied by individuals who smoke^8^. Our methodological approach was innovative and our data are novel in several regards. This is one of the first studies to explore correlates of individuals’ contamination with THS. Higher reported exposure to SHS/THS in friends’ and family members’ homes resulted in greater nicotine found on participants’ fingers. Further an important methodological innovation of this work included accounting for finger surface area. Indeed, using the median blank’s percentile as a modeling threshold, it appeared that larger finger area was significantly associated with higher nicotine levels. This variable accounted for the most variance at the highest percentile modeled as well (i.e. tau=0.774 associated with 2× median blank [>0.754 ng/wipe]), but was not statistically significant. Future studies should consider measuring finger (or hand size) for similar area calculations.

Being a non-smoker may not fully protect infants and children from THS. Being aware of smoking environments, including homes of individuals who report smoking outdoors^22,27,33^, and minimizing these exposure opportunities may prove essential for individuals seeking to eliminate THS exposure to the greatest extent. Importantly, common cleaning methods often fail to remove nicotine that adsorbs to indoor surfaces such as dust, doors, upholstery, pillows, mattresses, clothes, and especially carpets and sheet rock walls^7,8,37,38^. Indeed, 80–90% of combusted cigarette nicotine adsorbs to indoor surfaces^10^. The levels of nicotine found in our sample of medical staff are most similar to levels found on the fingers of non-smoking occupants staying in hotels with complete smoking bans^26^.

Our previous research indicates that a quarter of NICU infants will be discharged to a home with at least one smoker^22^, and non-staff visitors who smoke or live with individuals who smoke are expected to be a greater source of nicotine contamination in the NICU^6^. Future research will report on this. Still, it is concerning that a high proportion of this overwhelmingly non-smoking sample of people who report banning smoking in their homes and cars at very high levels (i.e. >93%) had measurable nicotine found on their skin. Medical staff were chosen because they have the greatest amount of patient contact; however, future studies should assess the degree to which front lobby staff, custodial, and other non-medical staff may contribute to nicotine contamination and this may aid to further quantify the cumulative degree of exposure for infants over NICU hospitalizations that may span weeks and months. Other hospitalized patients may also bring THS compounds to the hospital, as highlighted by a recent study of nicotine on the hands of pediatric patients who presented to an emergency department for SHS-related respiratory illnesses^39^.

This exploratory work was not without limitations. For example, we only measured one type of THS contamination (i.e. finger nicotine levels) and staff who refused may have been more exposed to THS. Future research should also account for nicotine found on clothing (e.g. scrubs) and personal items (e.g. mobile phones) brought to the NICU by staff. Additionally, we may have omitted several important variables related to nicotine contamination (e.g. hand washing frequency) due to the need to be highly efficient in sampling dozens of staff in 5- to 10-minute meetings. It is worth noting that no differences were found between finger-nicotine levels of staff measured at the beginning, middle, or end of a shift, suggesting that handwashing/scrubbing does not completely remove nicotine on skin, as shown elsewhere^40^. It is possible that nicotine (embedded within the skin) ‘sweats’ back to the surface.

The discovery that the majority of staff (78.3%) had detectable nicotine on their hands will be enlightening to hospital administrators and medical directors concerned about and interested in reducing potential contamination. Occasionally contacting and transporting nicotine through everyday activities (e.g. touching door handles, pushing shopping carts) would be impossible to avoid. However, asking staff about time spent in the homes of friends and family members who smoke may serve as a convenient proxy for determining higher risk of contamination caused by medical staff. While perhaps premature at this time, practical policy suggestions to reduce nicotine contamination may eventually entail increased handwashing frequency, storing/laundering hospital uniforms/scrubs in smoke-free hospital environments, and limiting the number of NICU visitors who smoke. Follow-on studies will continue to address these questions with medical staff and non-staff visitors.

## CONCLUSIONS

Infants who will reside with household smokers after NICU discharge will encounter high levels of THS, even if the home bans indoor smoking^33^. There is no safe level of SHS exposure, and due to the significant carcinogenic overlap between SHS and THS, it is likely that there is no safe level of THS exposure for hospitalized infants, many of whom may be immunocompromised and all of whom are undergoing respiratory development. THS exposure should be minimized to the greatest extent possible, particularly during a critical period such as the first few postnatal days, weeks, and months.
